# Awareness and attitudes of oncology specialists toward dihydropyrimidine dehydrogenase testing in Saudi Arabia

**DOI:** 10.1002/cnr2.1704

**Published:** 2022-08-14

**Authors:** Hatouf H. Sukkarieh, Turki AlSagoor, Mohammed Alnuhait, Rami Bustami, Scott Bryson, Fatima Mohammed Kebir Adem, Hana Abdalla, Gulsan Karbani

**Affiliations:** ^1^ College of Medicine Alfaisal University Riyadh Saudi Arabia; ^2^ College of Business Alfaisal University Riyadh Saudi Arabia; ^3^ Saudi Food and Drug Authority (SFDA) Riyadh Saudi Arabia; ^4^ College of Pharmacy, Department of Clinical Pharmacy Umm Al Qura University Makkah Saudi Arabia; ^5^ Institute of Pharmacy & Biomedical Sciences University of Strathclyde Glasgow Scotland

**Keywords:** 5‐fluorouracil, capecitabine, dihyropyrimidine dehydrogenase deficiency, fluoropyrimidines, personalized medicine, pharmacogenomics

## Abstract

**Background:**

Fluoropyrimidines (FP) are among the most common class of prescribed anti‐neoplastic drugs. This class has severe to moderate toxicity in around 10%–40% of those who take 5‐fluorouracil (5‐FU) or capecitabine for the treatment of cancer. In practice many patients with severe toxicities from FP use had dihydropyrimidine dehydrogenase (DPD) enzyme deficiency. Several studies have proposed DPD screening before treatment with 5‐fluorouracil (5‐FU) and capecitabine or other drugs belonging to the FP group. This study aims to assess the level of awareness and attitudes of oncology specialists in Saudi Arabia toward genetic screening for DPD prior to giving FP. This highlights the importance of health guidelines required for implementation in our health care system, as a framework to adopt testing as a regular practice in clinical care. Based on the findings in this study, guidelines have been suggested for the Middle East North Africa region.

**Methods:**

A cross‐sectional survey study was conducted during 2021 targeting oncologists and clinical pharmacists working in the oncology departments across Saudi Arabia.

**Results:**

A total of 130 oncologists and pharmacists completed the questionnaire representing a response rate of 87%. Most of the respondents indicated that they prescribe FP in clinical practice, but 41% of respondents reported that they have never ordered a specific molecular test during their practice. Only 20% of respondents reported that they often screen for DPD deficiency prior to prescribing FP. Significantly higher rates of awareness of potential dihydropyrimidine dehydrogenase gene (DPYD) mutation were observed among respondents in governmental hospitals (81.1% vs. 47.4% in private hospitals), and among those with more years of practice (80.6% if 5 or more years of practice vs. 59.3% if less than 5 years of practice). Also, higher rates of observing the impact of DPD testing were present among respondents with a PharmD (35% vs. 11% for oncologists and 18% for other professions) and among those with 5 or more years of practice (24.6% vs. 7.7% among those with less than 5 years).

**Conclusion:**

While in some institutions there is a high level of awareness among oncology specialists in Saudi Arabia regarding the effect of the potentially serious DPD enzyme deficiency as a result of gene mutations, screening for these mutations prior to prescribing FP is not a routine practice in hospitals across the country. The findings of this study should promote personalized medicine with recognition of interpatient variability via DPD testing to manage the risks of FP prescribing more effectively in the Kingdom of Saudi Arabia.

## 1 INTRODUCTION

Fluoropyrimidines (FP) are considered among the most highly prescribed chemotherapeutic agents which are commonly used in clinical practice. They include 5‐fluorouracil (5‐FU) and its prodrugs (capecitabine and tegafur).^1^, ^2^ This class of drugs is used for certain types of cancer such as head, neck, colorectal, pancreatic, and breast cancer.^1^ FP act via different mechanisms of action mainly by inhibiting thymidylate synthase (TYMS), and by incorporating metabolites into the cellular DNA and RNA.^2^ FP are associated with many adverse events such as severe diarrhea, mucosal ulceration, neutropenia, neurotoxicity, and hand‐foot syndrome also defined as palmar‐plantar erythrodysesthesia.^3^ The severity of toxicity increases when the patients have varying degrees of dihydropyrimidine dehydrogenase (DPD) deficiency. This can lead to life threatening toxicity following treatment with standard doses.^3^

Eighty percent of the administered FP is metabolized by the DPD liver enzyme.^4^ DPD has a physiological role in the breakdown of thymine and uracil in the body. This enzyme also plays a key role in the metabolism of the chemotherapeutic agents 5‐FU and capecitabine. Having a deficiency in DPD enzyme markedly increases the risk of severe and potentially life‐threatening toxic reactions.^2^

DPD deficiency is an inherited condition characterized by a wide range of symptoms; some patients experience neurological problems from infancy while no signs or symptoms are evident in others.^5^ This highlights the challenge for oncology specialists (medical, pharmacist, and nursing) to anticipate patients who may be at risk of severe toxicity from FP prescribing. There may be no clinical warning signs as some patients may be completely asymptotic.

About 2%–8% of the general population may be vulnerable to toxic reactions to FP drugs caused by asymptomatic DPD deficiency.^6^ Four genetic variants are currently considered clinically actionable: c.1905+1G>A, c.1679T>G, c.2846A>T, and c.1236G>A/HapB3. These four variants form the foundation of the current pharmacogenetic guidelines. However, these studies were based on clinical findings conducted in populations of limited diversity, mainly of European descent (Caucasians). This might explain the lower allele frequency of the four mentioned variants in our Saudi population (Saudi Human Genome Project Database).^3^, ^7^ According to the Clinical Pharmacogenetics Implementation Consortium (CPIC®), decreasing the FP dose may help to overcome a partial deficiency of DPD enzyme. However, in the case of complete deficiency, an alternative drug is a safer option.^3^, ^8^ Both partial and complete DPD deficiency are associated with increased risk of morbidity and mortality.^9^, ^10^, ^11^

The national guidelines for colorectal cancer in the Netherlands recommend DPD testing prior to therapy.^12^ It is highly recommended by the European Medicines Agency (EMA) and the UK Medicines and Healthcare Products Regulatory Agency (MHRA) to screen patients prior to giving these medications.^13^, ^14^ However, in Saudi Arabia, awareness and attitudes of healthcare professionals toward DPD testing with FP have yet to be established. The aim of this study was to assess the awareness and perceptions of oncology specialist healthcare providers (HCP) to screen for DPD testing before prescribing FP in Saudi Arabia. The specific objectives are: (1) to evaluate the level of HCP (oncologists and clinical pharmacists), overall and by their demographic characteristics (age, hospital department, duration of work experience and current designation), performing DPD screening tests before giving FP to cancer patients in Saudi Arabia, and (2) to examine the prevalence and application of DPD testing and Pharmacogenomics in Saudi Arabia.

## 2 DATA AND METHODS

This was a cross‐sectional study where a survey was designed and distributed to oncologists and clinical pharmacists in different hospitals in Saudi Arabia. The questionnaire was adapted and modified from previous studies.^15^ The survey included 26 items addressing the following topics: for example, participant demographics, awareness of oncologists and clinical pharmacists toward DPD screening. The content validity of the questionnaire items was conducted with the oncologists toward DPD testing. This was established by experts (oncologist and pharmacist) who examined the appropriateness of the content after making necessary modifications to the items to ensure they were comprehensive and accurately assessed and measured attitudes.

The questionnaire was distributed to selected HCPs who worked in oncology centers and hospitals with support from the Saudi Commission for health specialties to target active registered practitioners in oncology.

Inclusion Criteria: All oncologist and clinical pharmacist in all cancer centers in Saudi Arabia.
Exclusion Criteria: Retail pharmacists, outpatient pharmacists and non‐oncology doctors.

### 2.1 Sample size calculations

Previous studies have estimated the prevalence of health care professionals (HCPs) awareness toward pharmacogenomics testing to be between 31% and 62%.^15^, ^16^, ^17^ For our study, we assumed that the awareness of DPD testing was ~45%. Assuming the desired precision level of 5%, a confidence interval of 95%, and a total of 162 registered practitioners in oncology, as reported by the Saudi Commission for Health Specialties, the current study required the enrollment of 114 oncologists to achieve this target.

### 2.2 Statistical analysis

Descriptive statistics were performed on data collected from the study sample. For continuous variables, data were expressed as means ± SDs, medians, and ranges. Proportions were used to describe categorical variables. The awareness was evaluated and compared by demographic characteristics including age, hospital department, duration of work experience and current designation using the chi‐square test. Statistical significance is defined as *p* < .05. All statistical analyses were performed using IBM SPSS 21.0 (Armonk, NY: IBM Corp).

## 3 RESULTS

**FIGURE 1:**
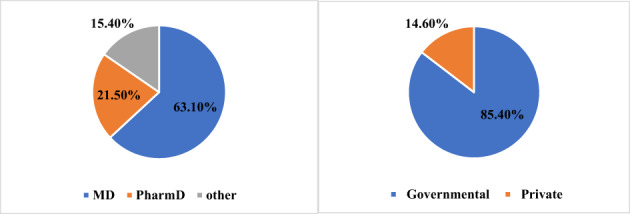
Descriptive statistics hospital type and last degree obtained

To account for an anticipated response rate of 75%, the questionnaire was administered to 150 oncology specialists, out of whom 130 completed it, indicating a higher than expected response rate of 87%. Descriptive statistics of the study sample are provided in Table 1 and Figure 1. The majority of respondents were aged 36 years or older (69%) and work in governmental hospitals (85%). The distribution of professions was as follows: 40.8% consultant, 17.7% clinical pharmacist, and 12.3% associate consultant, 10.8% oncology clinical pharmacist, 9.2% assistant physician, and 9.2% other professions. In relation to qualifications, 63.1% were MD, 21.5% PharmD, and 15.4% other. The distribution of years of practice was as follows: 20.8% less than 5 years, 18.5% 5–10 years, and 60.8% more than 10 years.

**TABLE 1:** Descriptive statistics of the study sample

Factor	Value
Age group (years) *n* (%)	
23–35	41 (16.7%)
36–45	60 (46.2%)
46+	29 (22.3%)
Profession	
Oncology consultant	53 (40.8%)
Clinical pharmacist	23 (17.7%)
Associate consultant	16 (12.3%)
Oncology clinical pharmacist	14 (10.8%)
Assistant physician	12 (9.2%)
Other	12 (9.2%)
Years of practice (%)	
<5	27 (20.8%)
5–10	24 (18.5%)
>10	79 (60.8%)
Years of oncology practice *n* (%)	
<5	65 (50.0%)
5–10	26 (20.0%)
>10	39 (30.0%)

*Note*: Total number of respondents = 130.

**FIGURE 2:**
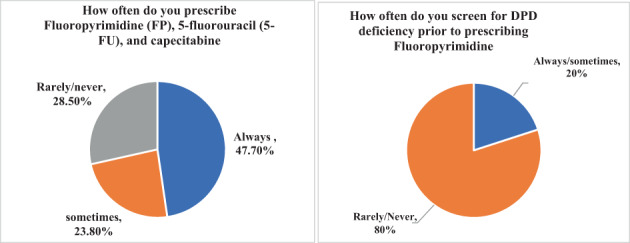
Existing practices (prescription, and screening)

Fifty‐nine percent of respondents reported that they have never ordered a specific molecular test during their practice (Table 2). The majority of respondents (76.2%) indicated that they always or sometimes prescribe FP, for example, 5‐fluorouracil (5‐FU), and capecitabine (Figure 2). Only 20% of respondents reported that they screen for DPD deficiency prior to prescribing FP.

**TABLE 2:** Existing practices and awareness and attitudes of oncologists toward DPD testing

Item	Option	No of responses	%
Have you ever ordered a specific molecular test during your practice?	Yes	53	40.8
	No	77	59.2
Are you aware of a mutation that causes dihydropyrimidine dehydrogenase deficiency (DPD)?	Yes	108	83.8
	No	21	16.2
Are you aware of the drug‐gene interaction caused by this mutation?	Yes	99	76.2
	No	31	23.8
Are there guidelines for prescribing fluoropyrimidines in your hospital?	Yes	50	38.5
	No	52	40.0
	I do not know	28	21.5
Do you think there is enough research done in Saudi on screening for DPD prior to prescribing fluoropyrimidines?	Yes	6	4.6
	No	72	55.4
	I do not know	52	40.0
Have you seen patients with severe toxicity related to use of fluoropyrimidines?	Yes	86	66.2
	No	44	33.8
Have you seen the impact of DPD testing in your clinical practice?	Yes	21	16.2
	No	109	83.8
Is there any standardization of dihydropyrimidine dehydrogenase (DPD) deficiency practice done by the ministry of health or your institute?	Yes	5	3.8
	No	62	47.7
	I do not know	63	48.5
Does the ministry of health or your institute promote the practice of dihydropyrimidine dehydrogenase (DPD) deficiency screening prior to giving fluoropyrimidines (FP) group of medications?	Yes	6	4.6
	No	66	50.8
	I do not know	58	44.6

*Note*: Total number of respondents = 130.

Eighty four percent and 76% of respondents reported awareness of a mutation that causes DPD deficiency, and drug‐gene implications (toxicity) that is caused by this mutation, respectively. Only 38.5% of respondents indicated the existence of guidelines for prescribing FB in their hospital, while 66.2% said that they have seen patients with severe toxicity related to the use of FP. Only 16.2% of respondents reported that they have seen the impact of DPD testing in their clinical practice. The vast majority of respondents (61.5%) did not know of any available guidelines or protocols in their own institute or by the ministry of health. Also, they noted that there is lack also of promotion of the practice of DPD deficiency testing before FP group of medications.

Table 3 shows survey results for the practices, awareness, and attitudes toward DPD testing by demographic and hospital‐related characteristics of the respondents. The rate of reporting the existence of guidelines for the treatment of hypertensive/non‐hypertensive diabetic patients was significantly different by demographic and practice‐related factors (hospital, profession, and years of practice [*p* < .05]). Significantly higher rates of awareness of the drug‐gene interaction causing mutation were observed among respondents working in governmental hospitals compared to private hospitals 81.1% versus 47.4% respectively (*p* = .003), and among those with more years of practice (5 or more 80.6% vs. 59.3% for those with <5 years; *p* = .021). In addition, significantly higher rates of observing the impact of DPD testing were observed among respondents with PharmD compared to those with MD and other degrees (35% vs. 11% and 17.9%, respectively; *p* = .031), as well as among those with more years of practice (24.6% vs. 7.7% among those with fewer practice years; *p* = .009).

**TABLE 3:** Awareness and observation of DPD testing in practice, by demographic and hospital related factors

Factor	*N*	Number with characteristic		%	*p* Value^a^
*Awareness of drug gene interaction causing mutation*					
Hospital type					.003
Governmental	111	90		81.1	
Private	19	9		47.4	
Last degree obtained					.060
MD	82	68		82.9	
PharmD	20	13		65.0	
Other	28	18		64.3	
Years of practice *n* (%)					.021
<5	27	16		59.3	
5 or more	103	83		80.6	
*Observation of the impact of DPD testing*					
Hospital type					.084
Governmental		111	15	13.5	
Private		19	6	31.6	
Last degree obtained					.031
MD		82	9	11.0	
PharmD		20	7	35.0	
Other		28	5	17.9	
Years of practice—Oncology *n* (%)					.009
<5		65	5	7.7	
5 or more		65	16	24.6	

*Note*: Total number of respondents = 130.

a Based on the chi‐square test or *t* test/Mann–Whitney *U* test.

## 4 DISCUSSION

FPs are a commonly used group of chemotherapeutic drugs in the management of several types of cancer.^6^ DPD testing can identify individuals with increased risk of severe toxicity to these drugs. Our study showed that the majority of oncology specialists were aware of these issues. Our study observed significantly higher rates of awareness of drug‐enzyme interaction causing mutation among respondents working in governmental hospitals (81% vs. 47% in private hospitals), and those with more years of practice (81% vs. 59% for those with fewer years of practice). However, guidelines to support FP prescribing were available to only 38.5% of respondents, only 20% indicated that they always or sometimes screen for DPD deficiency prior to FP prescribing, and only 4.6% of clinicians reported that their institutions promoted DPD screening prior to FP prescribing. This implies unexpectedly low uptake of a screening test which is now considered routine in many other countries. Therefore, our findings highlight the need for promoting DPD screening prior to administering FP.

Only 20% of the respondents in the study reported that they screen for the DPD variant prior to administering FP. Regardless, about 80% of the professionals are aware of DPD and the potential interaction with anti‐cancer drugs, while most of the respondents were aware of the presence of different DPD variants that might affect the level of FP toxicity.

Many countries worldwide have specific guidelines for FP therapy.^12^ Recent studies have shown that the cost of managing patients with severe toxicity is higher than the cost of the prospective DPYD testing of each new patient commencing FP treatment. These results therefore showed that tests for the detection of DPYD variants is a cost‐effective strategy.^18^

The authors of this report aspire to help in establishing a national clinical guideline addressing DPYD screening and genetic counseling prior to FP‐based chemotherapy. EMA and MHRA recommend testing all patients for DPD deficiency prior to systemic treatment with FP drugs. Patients with a known complete DPD deficiency must not be given FP. As for patients with a partial DPD deficiency, a reduced starting dose should be considered.^13^, ^19^ The EMA's recommendations have been adopted by the Federal Institute for Drugs and Medical Devices in Germany, and other scientific medical associations including United Kingdom, Germany, Austria, and Switzerland have developed proposals for implementing EMA recommendations.^2^ Moreover, the Dutch Pharmacogenetics Working Group (DPWG) published guidelines, which concluded that DPYD genotyping is considered “essential” and has directed DPYD testing as a requirement prior to initiating FP.^5^

An interesting study reported that HCPs have limited awareness about personalized medicine in general, but have positive attitudes toward it and are keen and eager to learn more about personalized medicine for future practice.^17^

In a further recommendation, additional training and awareness programs are required to raise health care providers' understanding of personalized medicine and to assist its implementation in Saudi Arabia. More emphasis on this topic should be put into the curricula of health colleges.^17^

The major strength of this study is its uniqueness in terms of assessing awareness of healthcare professionals about genetic screening prior to administering FP drugs in Saudi Arabia. We used an instrument that was carefully developed to examine physicians' current practice and attitudes toward such screening. We also explored the association between the demographic characteristics and awareness and attitudes of oncologists toward DPD testing in Saudi Arabia.

A limitation of this study is its reliance on participants' capacity to recall prior experiences, which might have resulted in bias. We limited recollection time to 1 year to reduce the possibility of such bias. Based on our findings, we recommend that immediate measures should be taken to increase awareness among oncology staff about genetic testing, and written guidelines related to FP should be established and implemented.

## 5 CONCLUSION

Our survey clarified the status of awareness of healthcare providers about performing genetic testing prior to FP. Genetic testing was not prevalent among most patients in Saudi Arabia. A lack of awareness regarding the existing rules and policies related to testing before FP was observed. The results of the study indicate an urgent need for actions to improve clinical practice by screening to find genetic variants for DPD deficiency prior to prescribing FP. A simple, inexpensive and cost effective test would substantially reduce the risk of serious and life threatening, drug related toxicity. Adoption of this practice will promote personalized medicine with safer medication use for FP in the Kingdom of Saudi Arabia.

## AUTHOR CONTRIBUTIONS

**Hatouf Husni Sukkarieh:** Conceptualization (lead); data curation (lead); formal analysis (lead); investigation (lead); methodology (lead); project administration (lead); resources (equal); software (equal); supervision (equal); validation (equal); visualization (equal); writing – original draft (equal); writing – review and editing (equal). **Turki Alsagoor:** Conceptualization (equal); data curation (equal); formal analysis (equal); investigation (equal); methodology (equal); project administration (equal); resources (equal); software (equal); validation (equal); visualization (equal); writing – original draft (equal); writing – review and editing (equal). **Mohammed Alnuhait:** Conceptualization (equal); data curation (equal); formal analysis (equal); investigation (equal); methodology (equal); resources (equal); software (equal); supervision (equal); validation (equal); visualization (equal); writing – original draft (equal); writing – review and editing (equal). **Rami Bustami:** Conceptualization (equal); data curation (equal); formal analysis (equal); investigation (equal); methodology (equal); resources (equal); supervision (equal); validation (equal); visualization (equal); writing – review and editing (equal). **Scott Bryson:** Conceptualization (equal); writing – original draft (equal); writing – review and editing (equal). **Fatima Adem:** Investigation (equal); methodology (equal); supervision (equal); validation (equal); visualization (equal); writing – original draft (equal); writing – review and editing (equal). **Hana Abdalla:** Conceptualization (equal); investigation (equal); methodology (equal); writing – original draft (equal); writing – review and editing (equal). **Gulsan Karbani:** Conceptualization (equal); investigation (equal); methodology (equal); visualization (equal); writing – review and editing (equal).

## CONFLICT OF INTEREST

The authors have stated explicitly that there are no conflicts of interest in connection with this article.

## ETHICS STATEMENT

Ethical approval was obtained from the Institutional Review Board (IRB) of Saudi Food and Drug Authority (SFDA), Research Center, Riyadh, Saudi Arabia. The IRB approval number is 04_2021. All participants signed a consent form prior to participation. The completed questionnaires were collected and securely stored in the principal investigator's office.

## Data Availability

The data that support the findings of this study is available from the corresponding author upon reasonable request.
